# The Future of Fracking: New Rules Target Air Emissions for Cleaner Natural Gas Production

**DOI:** 10.1289/ehp.120-a272

**Published:** 2012-07-02

**Authors:** Bob Weinhold

**Affiliations:** Bob Weinhold, MA, has covered environmental health issues for numerous outlets since 1996. He is a member of the Society of Environmental Journalists.


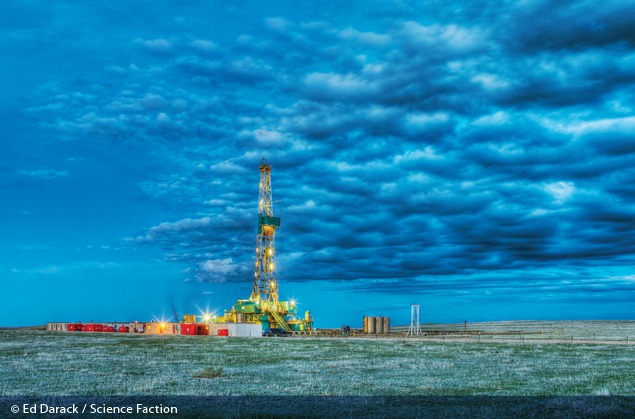
Natural gas is lauded as a cleaner-burning fuel than either coal or oil, but getting the fuel out of the ground can be a dirty process, especially given the widespread adoption of the technology known as hydraulic fracturing (“fracking”). Concerns about toxic air emissions from previously unregulated fracking sites led to the U.S. Environmental Protection Agency (EPA) announcement on 18 April 2012 of new and updated air pollution regulations for these facilities and certain other elements of oil and natural gas production and transmission.^1^ Compliance with the new regulations is expected to result in major reductions in emissions of methane and volatile organic compounds (VOCs), particularly from new fracked natural gas wells.

The rules were a hot topic nationally, drawing more than 156,000 comments after the proposed version was released in mid-2011. Under the final rules, companies have until January 2015 to fully phase in the control measures needed; by comparison, the initial proposal called for a 60-day phase-in for many major requirements. The EPA says about half of all new wells already use the equipment needed to capture the targeted emissions.^2^

**Figure:**
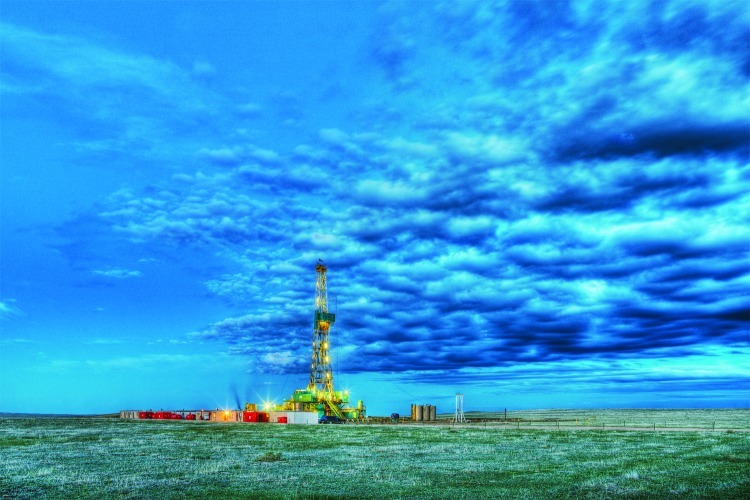
A hydraulic fracturing natural gas drilling rig on the eastern Colorado plains. In 2009 there were more than 38,000 natural gas wells in the state. © 2012 Ed Darack/Science Faction

Many environmental groups consider the new regulations an improvement over the existing situation, but they tend to be disappointed much more wasn’t done. “This is quite a milestone,” says Jeremy Nichols, Climate and Energy Program director for the advocacy group WildEarth Guardians, one of two groups that filed suit against the EPA in 2009 to force action on the issue. “But is the work done? No, of course not. It’s a floor to build on, providing a minimal level of protection.”

The oil and natural gas industry has its own concerns about the new rules but has indicated it can work with them. In a press release issued the day the rules were announced, Howard Feldman, director of regulatory and scientific affairs for the American Petroleum Institute, said, “EPA has made some improvements in the rules that allow our companies to continue reducing emissions while producing the oil and natural gas our country needs.”^3^

## Extraction in the United States

Oil and natural gas drilling are getting easier in some ways, as success rates for finding reserves have increased from 75% in 1990 to 90% in 2009. But companies must drill deeper to extract the resources, with oil and gas drilling depths steadily increasing from averages of 4,841 feet in 1990 to 6,108 feet in 2009. Fracking enables drillers to liberate hard-to-reach oil and hydrocarbons from underground deposits. Nevertheless, average natural gas productivity per well, measured in volume, steadily declined by a total of 36% between 1990 and 2009, with oil wells following suit with a drop of 17%.^4^^(Tables 2-4, 2-5, 2-6)^

In 2009 there were an estimated 1.02 million onshore oil and natural gas wells in the United States, split roughly evenly between the two types.^4^ The total is expected to steadily increase by about 17,000–35,000 natural gas wells and 9,000–10,000 oil wells per year between 2012 and 2035.^4^^(Table 2-13)^ Connecting the wells, processing plants, distribution facilities, and customers are more than 1.5 million miles of pipelines.^4^^(Table 2-8)^

A number of primary and secondary pollutants are linked with this web of facilities.^4^ One of them, methane, is over 20 times more potent a greenhouse gas than carbon dioxide (CO_2_) when emitted directly to the atmosphere.^5^ Hydrogen sulfide and VOCs such as benzene, ethylbenzene, toluene, mixed xylenes, *n*-hexane, carbonyl sulfide, ethylene glycol, and 2,2,4-trimethylpentane are classified by the EPA as hazardous air pollutants, or air toxics.^6^ Sulfur dioxide, nitrogen oxides, carbon monoxide, fine particulate matter (PM_2.5_), and ground-level ozone are classified as criteria air pollutants.^7^ Both classifications of pollutants cause adverse human health effects, but whereas criteria air pollutants are regulated by air quality standards that localities must achieve, hazardous air pollutants are regulated by requiring specific control technologies for the targeted emissions.

Among human health effects that have been associated with these pollutants are cancer; cardiovascular, respiratory, neurologic, and developmental damage; and adverse outcomes such as premature mortality, emergency department visits, lost work and school days, and/or restricted activity days. The pollutants are also associated with reduced visibility, climate change, and/or vegetation damage.^4^^,^^9^

Oil and natural gas production is the United States’ largest industrial source of VOCs, although a smaller source than the nation’s leading overall contributor, gasoline-powered vehicles.^8^ The industry also emits nearly 40% of the nation’s total methane.^4^ In 2015, even with the new rules in place, the oil and natural gas industry’s total VOC emissions will fall by only about 15% and its total methane emissions by only about 13%, according to figures provided by an EPA spokeswoman who spoke on condition of anonymity.

**Figure:**
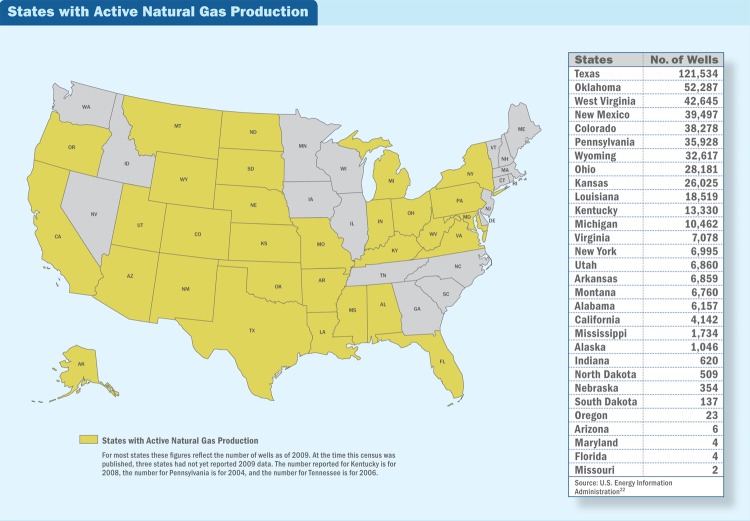
**States with Active Natural Gas Production**

In some cases, elevated concentrations of pollutants—some of them exceeding existing standards—have been documented around oil and natural gas facilities in states such as Wyoming,^10^^,^^11^ Utah,^10^ Colorado,^12^ New Mexico,^12^ and Texas.^13^ In many other cases, however, the concentrations of pollutants around these facilities are unknown.

In May 2012 the EPA designated a number of settings around the country as violating the 2008 ground-level ozone standard of 75 ppb—these included Bakersfield, California; Jamestown, New York; multicounty regions around Denver, Dallas, Fort Worth, Pittsburgh, Columbus, and Cleveland; and three counties in southwestern Wyoming. Many of these areas happen to host oil and natural gas operations, but many also have long histories of poor air quality related to other industries, making it difficult to tease out the contribution of oil and natural gas operations. The natural gas boom region of northeastern Utah also is suspected of contributing to local elevations in ground-level ozone, although there aren’t enough data for a formal violation designation.^14^

Under the Clean Air Act, the EPA is required to review certain regulations every eight years and revise them if necessary. These regulations include New Source Performance Standards, or NSPSs (which apply to specific types of newly built, modified, and reconstructed facilities), and National Emission Standards for Hazardous Air Pollutants, or NESHAPs (which apply to the air toxics emitted from various facilities). The NSPSs applicable to oil and natural gas production had not been updated since 1985, and the applicable NESHAPs had not been updated since 1999. So on 14 January 2009 WildEarth Guardians and fellow advocacy group San Juan Citizens Alliance filed suit to force the agency to act. The parties signed a consent decree 5 February 2010. The EPA issued proposed rules 28 July 2011 and signed the final regulations 17 April 2012.^15^

## A New Era

Some of the rules begin to take effect 60 days after they’re published in the *Federal Register* (which had not yet occurred as this article went to press), with various phase-in periods for other parts of the rules up to 1 January 2015. The rules apply to all relevant onshore facilities that have been constructed, reconstructed, modified, or refracked since 23 August 2011. The main focus of the new rules is most types of new fracked natural gas wells.^16^

The primary tool for controlling the relevant emissions is equipment that captures and separates the mixed gases, liquids, and other substances that flow from new wells. Completing the well installation process with this kind of pollution-control equipment has been dubbed a “green completion.” Much of the captured material includes resources with substantial market value, including propane, butane, and liquefied natural gas.^4^

**Figure:**
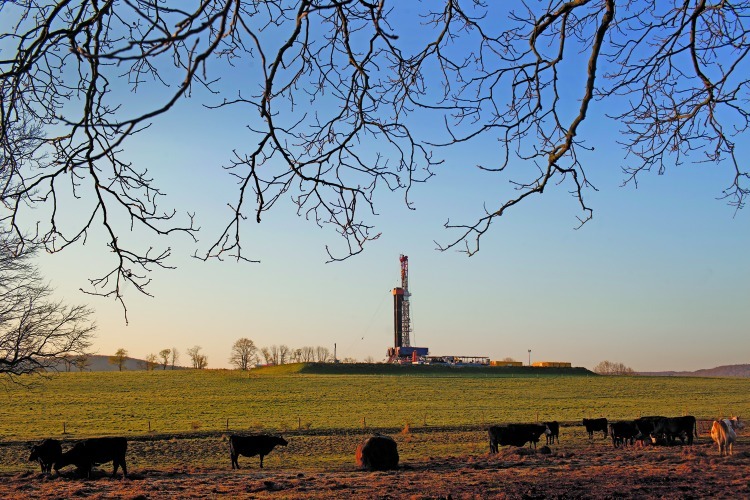
**Steady Growth in Natural Gas** The number of natural gas wells nationally has steadily risen from about 269,000 in 1990 to nearly 500,000 in 2010.4(Table2-5) Meanwhile, total oil production has steadily dropped since 1970 and is now at about two-thirds that peak, although there has been an uptick in the past couple of years, driven almost entirely by the oil fracking surge in North Dakota.^31^ The overall increase in natural gas extraction is being driven in large part by the increase in consumption, which rose 19% between 1990 and 2009.4(Table 2-10) Most of that increase occurred in the electric power sector, with its share of total consumption rising from about 17% in 1990 to about 30% in 2009. Industrial consumption has declined from about 43% of the total in 1990 to about 32% in 2009. Other sectors have remained fairly steady, with residential use at 20–24%, commercial use at 13–14%, and transportation at 3%.4(Figure 2-5) © 2012 Les Stone/Corbis

Green completions are mandatory for new wells beginning 1 January 2015 and are encouraged on a voluntary basis before that. Larger companies tend to be the ones already using green completions, Feldman says. In some cases, companies have opted not to use green completions because the necessary transportation facilities (e.g., pipelines for the various gas constituents) are not in place, he says. In other cases, he adds, low pressure in a well has made capture more difficult, or capture is less cost-effective when VOC content is low. Feldman says the 2015 implementation date will allow the industry enough time to get necessary infrastructure in place.

One company that has been using green completion equipment for more than half a dozen years is Devon Energy, headquartered in Oklahoma City. “It’s the right thing to do,” spokesman Chip Minty says. “It reduces emissions and keeps gas in the pipeline. And [the captured] commodities are just as valuable as any commodity from any well,” with no unusual impurities reducing their value.

Owners and operators that choose not to use green completions prior to January 2015 must burn off (or flare) the emissions coming from the new well. Flaring creates combustion pollutants such as carbon monoxide, nitrogen oxides, PM_2.5_, and CO_2_, and contributes to formation of often-uncharacterized secondary compounds. However, the EPA estimates that the benefits of preventing the escape of VOCs and methane far outweigh the damage caused by the pollutants produced by flaring.^4^ Gwen Lachelt, director of the Oil and Gas Accountability Project of the nonprofit Earthworks, says allowing flaring in transition is “certainly not ideal,” in part because it continues to waste valuable resources, but is an improvement over straight venting.

**Figure:**
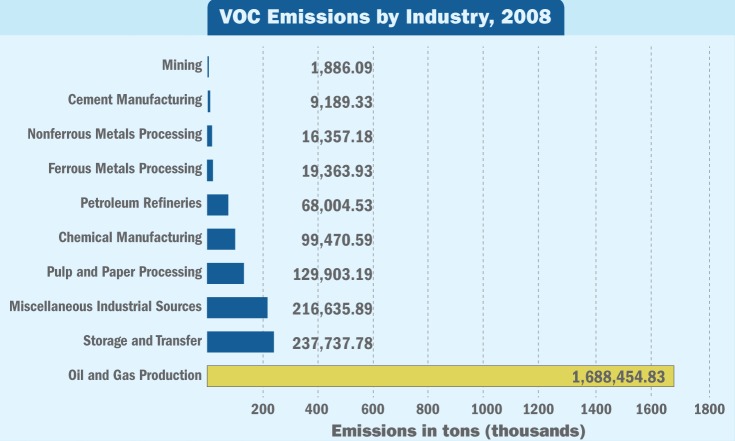
**VOC Emissions by Industry, 2008**

**Figure:**
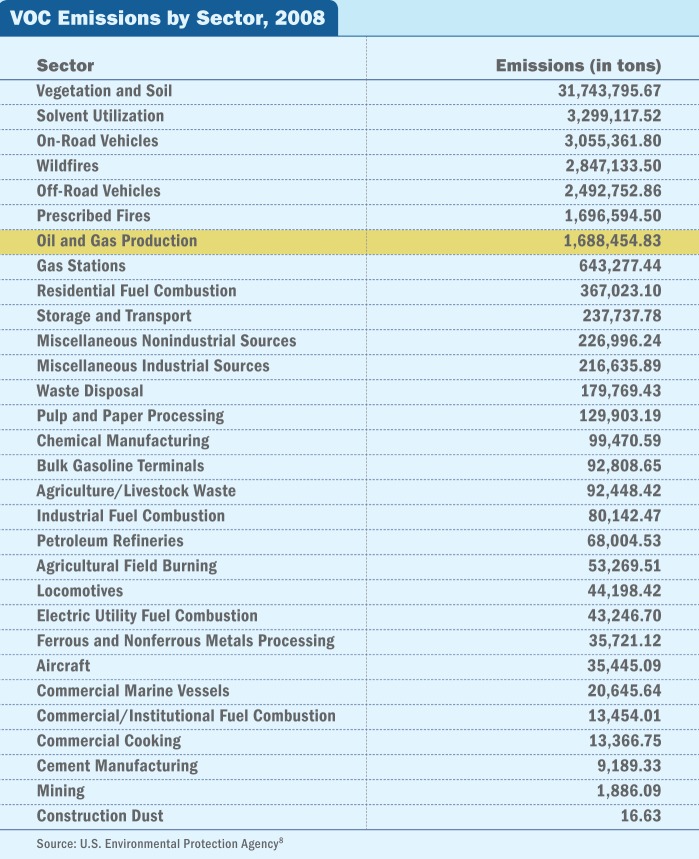
**VOC Emissions by Sector, 2008**

Finally, the new rules require reductions in emissions from equipment such as processing plants, storage tanks, pneumatic controllers, glycol dehydrators, and certain pipeline compressors, and they also add various reporting and notification requirements for the industry. “We find [the latter] to be extremely burdensome,” says Kathleen Sgamma, vice president of government and public affairs for the Western Energy Alliance, a nonprofit trade association. “It’s a lot of new record keeping with not a lot of additional environmental benefit.” Nichols of WildEarth Guardians has a different view, saying the requirements could have been more stringent. “But they’re workable for information and transparency,” he says, which is “incredibly important so we can scrutinize if industry is complying.”

The EPA estimates the green completion process and other required changes will annually cut about 95% of the VOCs emitted from 11,400 newly fracked and 1,400 refracked wells.^17^ For 2015 the agency estimates that full implementation of the new rules will result in reductions of 190,000 tons of VOCs, 11,000 tons of hazardous air pollutants, and methane equivalent to 18 million tons of CO_2_ above and beyond reductions already mandated in Wyoming, Colorado, and a few places in Texas.^4^^,^^18^

The agency couldn’t calculate how much hazardous air pollutants as a whole will be reduced in the context of emissions from the total oil and natural gas industry. The agency also couldn’t calculate the reductions in pollutants such as hydrogen sulfide and criteria air pollutants PM_2.5_ and ozone. Nor could it estimate the dollar value of health benefits attributable to the rules because of uncertainties over exactly where future extraction operations would occur and what the local and regional impacts would be.^4^

However, after comparing the direct cost to industry of complying with the rules against profits from the sale of captured resources, the agency says the industry should net $11–19 million per year.^17^ Sgamma says that works out to “a miniscule amount” of about $900–1,500 possible profit per well. Upon full implementation the agency also estimates net annual climate-related benefits of about $440 million based on effects such as avoided adverse health effects and damage to crops and coastal property.^4^

**Figure:**
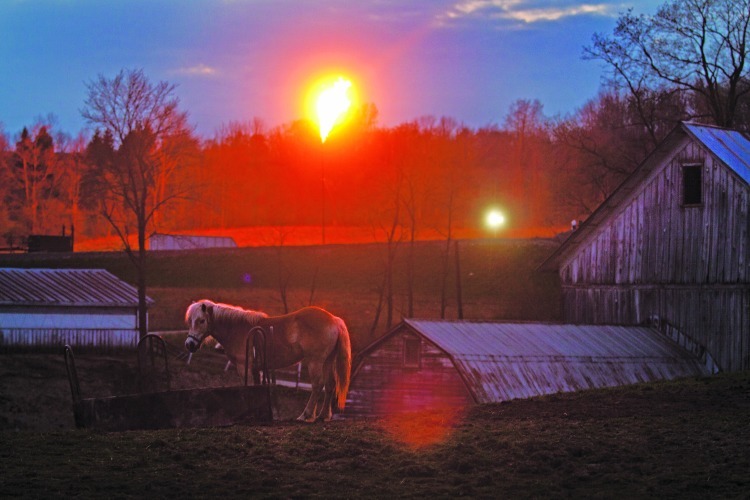
Flaring at a fracked natural gas well in Bradford County, Pennsylvania. Under the new EPA regulations, producers may either flare emissions from new wells until 2015 or capture the emissions using the green-completion equipment that will become mandatory for new wells starting in that year. Although cleaner than straight venting, flaring produces pollutants of its own and burns up valuable commodities. © 2012 Les Stone/Corbis

Feldman and Sgamma (among others^19^) say the EPA’s economic assessment is inaccurate, due to factors such as overestimating the quantities of sellable resources recovered and underestimating costs to industry. On 4 June 2012 the American Petroleum Institute and fellow industry association America’s Natural Gas Alliance released estimates of industry methane emissions that were half as much as the EPA estimated.^20^ The EPA spokeswoman says the agency will review the new report.

Industry expenses vary over time with market cycles, but perhaps the biggest variable is the future price of dry natural gas (nearly pure methane that has been processed to remove water and “wet” hydrocarbon gases that may accompany it out of the ground). The price has fluctuated fourfold between 1990 and mid-2012, often making major moves up and down in just a few years.^21^ The EPA based its economic calculations on numbers in the middle of this overall price range.^4^

## State-Level Actions

Oil and natural gas drilling occur in 33 states.^22^ The number could conceivably increase; North Carolina is aggressively working to see if recent developments in fracking technology might allow its small deposits, previously considered economically marginal, to become cost-effective.^23^ Vermont, which also has no producing wells at the moment, is taking a different approach, banning fracking until at least 2016 in order to study potential public health and environmental impacts and develop guidance for regulating the practice.^24^

As awareness of air pollution from natural gas extraction, processing, and transmission has risen, high-production areas such as the city of Fort Worth and the states of Wyoming and Colorado have begun requiring processes similar to green completions. Wyoming has also been monitoring some pollution hot spots, requiring some industry reporting of emissions, and revising its regulations, says Steven Dietrich, administrator of the Wyoming Department of Environmental Quality’s Air Quality Division. By 2015 he expects the state’s rules will be nearly identical to those of the EPA.

However, that alone won’t be enough to bring Wyoming counties currently violating the ground-level ozone standard into compliance. That job might have been easier if the new EPA rules had addressed existing wells and facilities. That exclusion “makes it more difficult to reduce more emissions,” Dietrich says, because Wyoming, like the EPA, is limited in its authority to rein in existing pollution sources. In the absence of EPA regulations, he says his department will implement strategies that have helped in the past, such as incorporating requirements for diesel-powered equipment into permitting processes.

In Arkansas, the state’s Department of Environmental Quality investigates pollutant leaks in the course of routine compliance inspections or in response to citizen complaints. The state utilizes new infrared cameras as a rapid-detection tool to document leaks, says Mike Bates, chief of the department’s Air Division. The department encourages companies to address leaks voluntarily but has the capacity to pursue enforcement if the company does not act.

Low levels of VOCs have been detected around drilling sites in Arkansas, which most likely came from tanks of diesel fuel–based drilling mud (a multipurpose fluid used in drilling boreholes). A 2011 report from the Arkansas Department of Environmental Quality states, “Although mud tanks are a temporary and probably minor emissions source, their emissions have a strong hydrocarbon odor that may be a nuisance and potential health risk to people living near well sites during the drilling process. Reducing VOC emissions from mud tanks may provide an opportunity to improve the local air quality around active drilling sites.”^25^

Other emissions have been relatively low in Arkansas compared with other natural gas–producing areas, although some important gaps in data remain, Bates says. This, he says, is likely in part because the gas extracted in Arkansas has low VOC content, and Southwestern Energy, the company that has more than three-fourths of the Arkansas market, already uses green completions extensively. That may make the state’s overall transition to the new EPA rules relatively painless for both the industry and the state. “Since a large segment of the industry is already meeting these standards, we don’t foresee a great impact to the regulated industry in complying with the new rules,” Bates says.

Pennsylvania, which lies atop the enormous Marcellus Shale Deposit, is just beginning to obtain hard data on its industry’s air emissions and will have a final inventory for submission to the EPA by December 2012. The state hasn’t conducted any long-term air monitoring focused on natural gas drilling activities but expects to begin doing so before the end of 2012. Short-term monitoring conducted in 2010 did not identify concentrations of any compound associated with natural gas drilling that would likely trigger air-related health issues, according to Pennsylvania Department of Environmental Protection secretary Mike Krancer, quoted in a December 2011 press release.^26^ The state is working on an updated set of permit requirements and is analyzing the EPA’s rules, says Kevin Sunday, a spokesman with the department. Senior department officials declined multiple requests to discuss the rules.

## Expanding the Base

The EPA explicitly chose to not have the new rules apply to existing wells because, on a per-well basis, new wells produce far more VOC emissions and can offset costs for implementing the new rules with sales of captured products. The fact that most existing oil and natural gas wells tend to have relatively low or unknown VOC emissions lessens the potential for applying the new rules to them in a cost-effective manner, even though, combined, they remain a major source of emissions of VOCs and many other pollutants.

Older facilities also can be sources of methane emissions. Based on recent legal developments, including a 2007 Supreme Court ruling and subsequent EPA efforts to regulate greenhouse gases as air pollutants,^27^ the EPA should have chosen to regulate methane directly, leading to an update of both methane and VOC regulations for all existing wells and facilities, says David Doniger, policy director for the Natural Resources Defense Council’s Climate and Clean Air Program. Since the agency didn’t take that path, Doniger says his organization is deciding whether to sue to force such action.

If they do, they’ll likely be challenged by the industry. “The EPA is using a VOC rule to pursue methane reduction in an almost backhanded way,” Feldman says. “That’s a concern.” But he acknowledges the agency may have the right to regulate methane as an air pollutant, although much litigation is still in process, and the U.S. Congress also could restrict such actions.^27^

Like Doniger, Lachelt is chagrined that existing wells and facilities weren’t addressed, from both a greenhouse gas and a hazardous air pollutant perspective. “We’re absolutely concerned about the impacts of these facilities on the health of people living near them,” she says. “To not include them [in the new rules] is tragic.”

Neither existing nor new fracked oil wells are covered by the new rules. That’s because “the EPA does not have sufficient data on VOC emissions during completion of hydraulically fractured oil wells to set standards for these operations at this time,” the EPA spokeswoman says. That allows the hundreds of thousands of new oil wells anticipated over the next 20-plus years to operate under existing rules if nothing changes. Much of the activity is likely to occur in high-production areas in North Dakota, California, Colorado, Kansas, Montana, Nebraska, New Mexico, Texas, and Wyoming, some of which began to surge in production in 2007.^28^ “This is a huge issue,” Nichols says. “We pushed the EPA to rope these in [to the new regulations], but they didn’t want to go down that road.”

Furthermore, according to Nichols, the new rules will not fully protect people against hazardous air pollutants even from new facilities, and they need to be made more rigorous to further reduce emissions. In concert with that, he’d like to see more stringent requirements for monitoring and repairing defects and leaks in pipelines. Some of the pipelines of most concern, according to a Government Accountability Office report issued in March 2012, are the so-called gathering pipelines that take natural gas from wells to processing facilities.^29^ Only about 10% of the 200,000 miles of gathering pipelines are regulated by federal or state agencies; the remainder tend to be more than 220 yards from human-occupied buildings, so regulation usually is waived.

Unregulated lines occur in at least 29 states. State pipeline safety officials canvassed by the Government Accountability Office say these lines are at elevated risk for poor construction quality, undetected corrosion, poor maintenance, and unmarked locations that increase the odds they will be hit when an area is excavated (which may occur more frequently as natural gas fields are developed close to urbanizing areas). All these problems can contribute to increased air pollution.^29^ So can the fallout from pipeline cyber attacks, which are an escalating concern at high levels of government.^30^

Despite these and other concerns acknowledged by some health and environmental advocates, industry members, and government officials, many agree the complex set of new EPA regulations are a decent start in the right direction. Dietrich says, “I thought the rules came out as well as can be expected, balancing the needs of all the different states.” Nichols also is looking positively at the overall result: “Clearly the final rules are a step away from what they initially proposed. Still, it’s a step forward.”
